# Structurally Similar Mycotoxins Aflatoxin B_1_ and Sterigmatocystin Trigger Different and Distinctive High-Resolution Mutational Spectra in Mammalian Cells

**DOI:** 10.3390/toxins17030112

**Published:** 2025-02-27

**Authors:** Pennapa Thongararm, Marisa Chancharoen, Nutchapong Suwanwong, Somsak Ruchirawat, Mathuros Ruchirawat, Bogdan I. Fedeles, Robert G. Croy, John M. Essigmann

**Affiliations:** 1 Laboratory of Environmental Toxicology, Chulabhorn Research Institute, Bangkok 10210, Thailand; mathuros@cri.or.th; 2 Department of Biological Engineering, Department of Chemistry, and Center for Environmental Health Sciences, Massachusetts Institute of Technology, Cambridge, MA 02139, USA; marisa@cgi.ac.th (M.C.); bogdan@mit.edu (B.I.F.); rgcroy@mit.edu (R.G.C.); 3 Program in Applied Biological Sciences, Chulabhorn Graduate Institute, Bangkok 10210, Thailand; 4 Program in Chemical Sciences, Chulabhorn Graduate Institute, Bangkok 10210, Thailand; nutchapongs@cgi.ac.th (N.S.); somsak@cri.or.th (S.R.); 5 Center of Excellence on Environmental Health and Toxicology (EHT), OPS, Ministry of Higher Education, Science, Research and Innovation, Bangkok 10400, Thailand; 6 Laboratory of Medicinal Chemistry, Chulabhorn Research Institute, Bangkok 10210, Thailand

**Keywords:** mycotoxins, high-resolution mutational spectra, mutagenesis, biomarkers, mammalian cells

## Abstract

Aflatoxin B_1_ (AFB_1_) and sterigmatocystin (ST) are mycotoxins that pose significant threats to human and animal health owing to their mutagenic, carcinogenic, and toxic properties. They are structurally similar and widely believed to exert their biological effects via the generation of DNA-damaging epoxides at their respective terminal furan rings. Despite structural identity in the warhead portion of each toxin, this work shows that distal parts of each molecule are responsible for the distinctive mutational fingerprints seen in *gpt*Δ C57BL/6J mouse embryo fibroblasts (MEFs). The two toxins differ structurally in the puckered cyclopentenone ring of AFB_1_ and in the planar xanthone functionality of ST. While both toxins mainly induce GC→TA mutations, the aforementioned differences in structure apparently trigger unique patterns of mutations, as revealed by high-resolution duplex sequencing of MEF genomes. AFB_1_ is more mutagenic than ST and displays its transversion mutations in a pattern with primary and secondary hotspots (underscored) in 5′-CGC-3′ and 5′-CGG-3′ contexts, respectively. ST displays a modest 5′-CGG-3′ hotspot while its other GC→TA transversions are more uniformly distributed in a pattern resembling established oxidative stress mutational spectra. This research delineates the mutational spectra of AFB_1_ and ST, establishing these patterns as possible early-onset biomarkers of exposure.

## 1. Introduction

Mycotoxins represent one of the most significant global threats to public health and food safety, with aflatoxin B_1_ (AFB_1_) and sterigmatocystin (ST) standing out as two toxicologically relevant examples [1,2,3,4,5,6,7,8]. Produced by several species of *Aspergillus* molds, these secondary metabolites have raised considerable concern due to their prevalence in agricultural products, particularly in areas of the world with inadequate food storage practices [9,10]. The urgency of addressing the risks posed by AFB_1_ and ST has been heightened by the effects of climate change. Warming temperatures and shifting environmental conditions have created favorable habitats for heat-tolerant *Aspergillus* species, leading to increased contamination of staple crops, including maize, peanuts, and grains [11,12]. These environmental changes disproportionately impact developing regions, where agricultural practices often lack the infrastructure for effective mitigation. Consequently, the contamination of food supplies by AFB_1_ and ST has become not only a food safety issue but also a major public health concern, contributing to long-term health risks associated with liver damage, cancer, and immune suppression [13,14]. Understanding the biochemical pathways and the genetic mechanisms underlying the toxicity of these compounds is therefore critical for developing effective early disease detection and preventive strategies, and appropriate regulatory frameworks.

**Figure 1:**
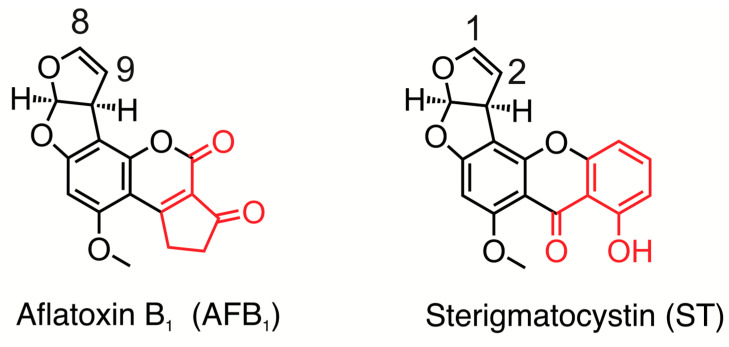
Comparison of the chemical structures of AFB_1_ and ST. Both molecules share a similar core structure (black) that includes a difuran moiety that has a double bond between the 8 and 9 carbons of AFB_1_ and the 1 and 2 carbons of ST. Differences between the structures of AFB_1_ and ST are highlighted in red.

AFB_1_ and ST (Figure 1) are mycotoxins synthesized via a shared biosynthetic pathway involving approximately 20 enzymatic steps [15]. Late in the pathway, the xanthone ring of ST is converted to the coumarin–cyclopentenone product, AFB_1_. Figure 1 has differential coloration to show the differences between the two toxins. Both toxins originate from a common polyketide precursor that undergoes enzymatic cyclization and modifications, culminating in the formation of the difuranocoumarin core [16,17]; this structural motif underpins many of their toxicological effects, as it facilitates bioactivation into reactive intermediates that can interact with cellular macromolecules [18,19]. Despite their structural similarities, AFB_1_ and ST exhibit distinct toxicological profiles, partly due to differences in their metabolic activation and interaction with cellular targets. AFB_1_ is bioactivated primarily by cytochrome P450 enzymes, specifically CYP1A2 and CYP3A4 in humans, to generate the highly reactive *exo*-AFB_1_-8,9-epoxide [19,20,21,22,23,24,25,26,27]. This epoxide intermediate forms covalent DNA adducts, most notably 8,9-dihydro-8-(N7-guanyl-9-hydroxyaflatoxin B_1_ (AFB_1_-N7-Gua) [20] and its imidazole ring-hydrolyzed derivatives, the AFB_1_ formamidopyrimidines (FAPYs) [27]. These DNA adducts disrupt normal base-pairing during replication, leading to mutations that can initiate carcinogenesis [4,21,28,29,30,31,32,33,34,35,36]. The mutational spectrum induced by AFB_1_ is well-characterized, with hallmark mutations frequently identified in the *TP53* tumor suppressor gene in hepatocellular carcinoma (HCC) patients [37,38,39]. Epidemiological evidence underscores the strong correlation between chronic AFB_1_ exposure and liver cancer, particularly in populations co-infected with hepatitis B virus, where a powerful synergistic interaction amplifies disease risk [40,41].

ST undergoes a similar metabolic activation, yielding the epoxide intermediate *exo*-ST-1,2-epoxide. This metabolite also forms DNA adducts, predominantly 1,2-dihydro-2-(N7-guanyl)-1-hydroxysterigmatocystin (ST-N7-Gua) [22,42,43], but its mutagenic effects appear to be less pronounced than those of AFB_1_ [44,45]. While the cytotoxicity and DNA-binding capacity of ST are well-documented [43,44,46,47], its mutational spectrum remains uncharacterized, complicating efforts to associate its mutagenic profile with possible downstream cancers. Regulatory agencies have responded to these uncertainties with varying levels of oversight, with AFB_1_ subject to strict limits, such as 20 µg/kg in food products set by the U.S. Food and Drug Administration [48] and even lower thresholds established by the European Union [49]. In contrast, ST is less stringently regulated, reflecting the limited data on its long-term health impacts.

Despite extensive research on AFB_1_, significant gaps remain in our understanding of its precise molecular mechanisms and its interactions with other environmental and genetic factors. Recent technological advancements have provided new tools for addressing these challenges. High-resolution DNA sequencing has emerged as a powerful approach for delineating the detailed context-dependent mutational patterns induced by mycotoxins. These patterns offer valuable insights into the molecular origins of cancer and provide a basis for comparing experimental models to human malignancies [34,50]. In parallel, bioinformatic analyses have enabled the identification of mutational signatures associated with specific environmental exposures, enhancing our ability to link experimental findings to epidemiological data [51]. In this study, we leverage these recent advances to investigate the cytotoxic and mutagenic effects of AFB_1_ and ST using mouse embryo fibroblasts (MEFs) derived from the *gpt*∆ C57BL/6J mouse, an animal model that is widely used in regulatory toxicology. This model, previously validated for studying DNA alkylation [50,52] and oxidative stress [53], provides a robust platform for exploring the mechanisms underlying mycotoxin-induced DNA damage. To account for the requirement of metabolic activation, we incorporate in the present work a microsomal activation system, enabling the formation of reactive intermediates. We also employ bioinformatic tools to compare the mutational patterns observed in our model to those found in human cancers, providing a translational link between experimental and clinical findings.

The findings from this study might have significant implications for public health and food safety. By elucidating the mechanisms through which AFB_1_ and ST induce DNA damage and mutations, we aim to provide a clearer understanding of their relative risks and inform strategies for mitigating their impact. Such strategies are particularly important in regions of the world where contamination of food by these toxins is pervasive and regulatory enforcement is limited. Improved detection methods, coupled with enhanced storage practices, could play critical roles in reducing exposure. Moreover, our work highlights the need for a more nuanced approach to the regulation of mycotoxins. While the stringent limits on AFB_1_ contamination reflect its well-documented risks, the relative lack of regulation for ST may underestimate its potential health impacts. A more comprehensive assessment of ST, informed by advanced toxicological and epidemiological studies, could provide the basis for updated regulatory guidelines.

## 2. Results

### 2.1. Metabolic Activation Distinguishes the Cytotoxicity Profiles of AFB_1_ and ST in MEFs

**Figure 2:**
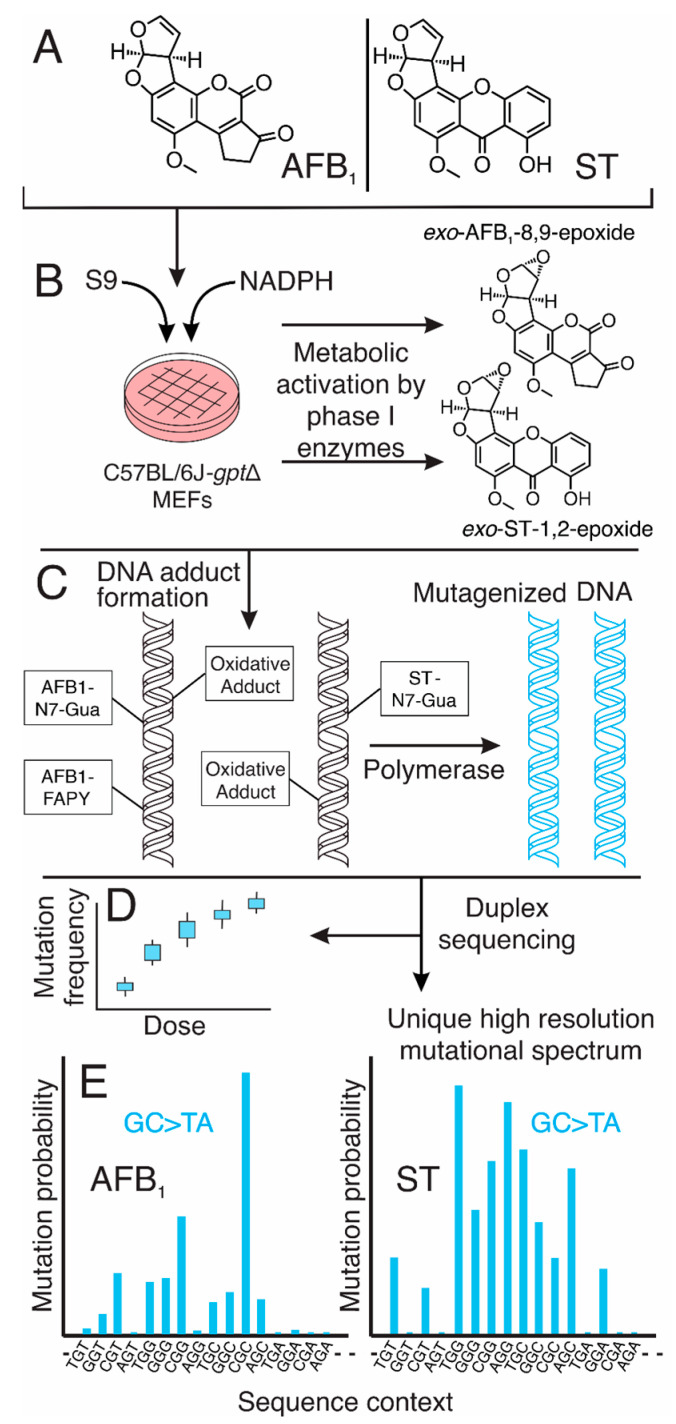
Schematic overview of experimental workflow for determining mutagenic signatures of AFB_1_ and ST. (**A**,**B**) The carcinogenic mycotoxins AFB_1_ and ST undergo metabolic activation to form highly reactive epoxide intermediates capable of covalently bonding to DNA. Mouse embryo fibroblasts derived from *gpt*Δ C57BL/6J mice were treated with AFB_1_ or ST in the presence of S9 metabolic activation and NADPH. Phase I metabolism converts the parent compounds into their respective electrophilic species: exo-AFB_1_-8,9-epoxide and exo-ST-1,2-epoxide. (**C**) Reactive epoxide metabolites form DNA adducts, such as AFB_1_-N7-Gua, AFB_1_-FAPY, and ST-N7-Gua, which disrupt DNA structure and informational integrity. These adducts lead to mutations following bypass by error-prone DNA polymerases or failed repair. (**D**) Mutational frequency was quantified across multiple toxin doses using a highly sensitive *gpt* assay. (**E**) High-resolution mutational spectra were generated using duplex DNA sequencing, enabling precise characterization of the mutation profiles induced by AFB_1_ and ST. GC→TA transversions emerged as the hallmark mutation type for both compounds, with distinct sequence-context dependencies reflected in unique mutational fingerprints.

**Figure 3:**
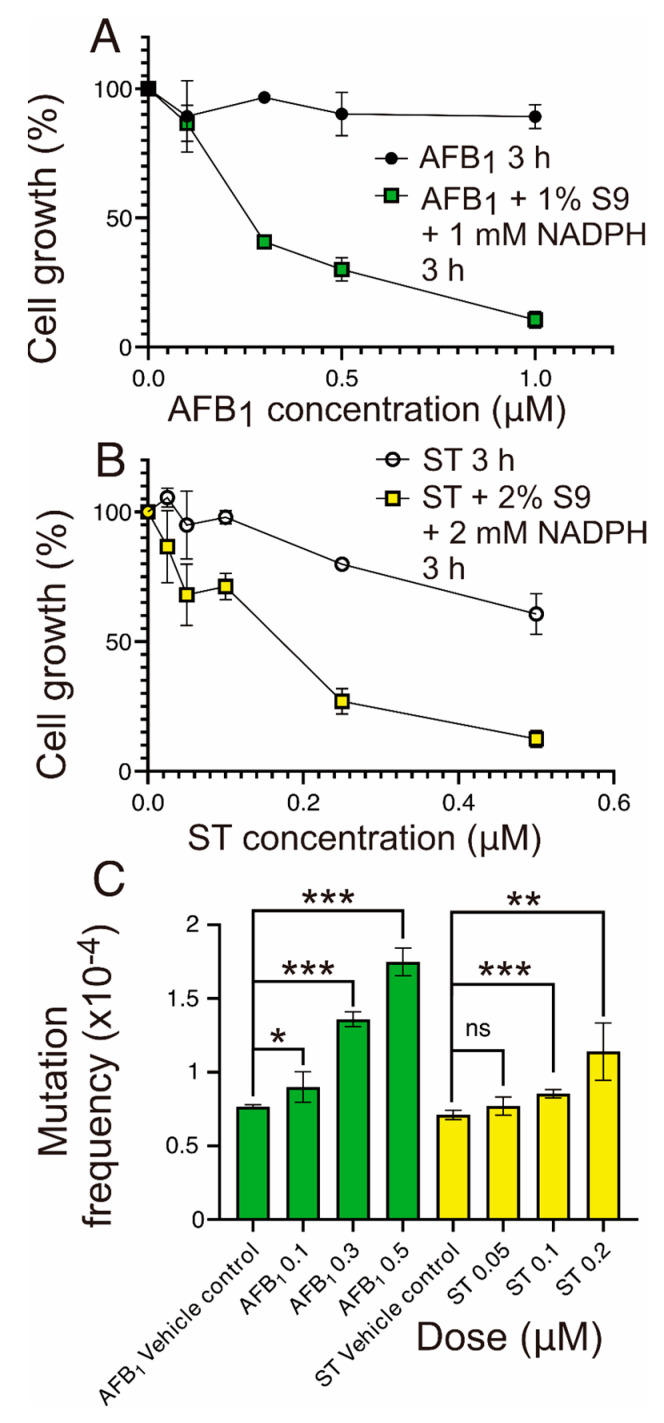
Cytotoxicity and mutagenicity of AFB_1_ and ST in MEFs with and without S9 metabolic activation. (**A**) Percent cell growth in MEFs treated with AFB_1_ for 3 h, depicted as a comparison between conditions without S9 (black circles) and with S9 metabolic activation (green squares; 1% S9 and 1 mM NADPH). (**B**) Percent cell growth in MEFs treated with ST for 3 h, shown without S9 (open circles) and with S9 metabolic activation (yellow squares; 2% S9 and 2 mM NADPH). (**C**) Mutation frequency induced by AFB_1_ and ST under S9 metabolic activation at selected concentrations, with dose-dependent effects evident for AFB_1_. Data are expressed as mean ± SD, with statistical significance denoted as * *p* < 0.05, ** *p* < 0.01, *** *p* < 0.001, and ns = not significant.

Figure 2 depicts the experimental workflow of this study. The cytotoxic effects of AFB_1_ and ST on MEFs were significantly influenced by metabolic activation, which substantially increased the toxicity of the toxins to the cell culture model (Figure 3A,B). Without metabolic activation, AFB_1_ exhibited minimal cytotoxicity, with cell viability exceeding 90% even at the highest concentration tested (1 µM). Interestingly, however, even without metabolic activation ST demonstrated enhanced dose-dependent baseline toxicity, making it more toxic than aflatoxin at any given dose. Upon activation with the S9 fraction and NADPH, both compounds showed markedly enhanced toxicity, but the effect was more pronounced for AFB_1_, which led to near-complete inhibition of cell growth at 1 µM. This result is in line with the expected conclusion that AFB_1_ strongly relies on enzymatic bioactivation to produce reactive metabolites responsible for triggering its potent cytotoxicity. While ST also became significantly more toxic following S9 activation, its intrinsic cytotoxicity in the absence of metabolism underscores fundamental differences between it and AFB_1_ in their toxicological profiles.

### 2.2. Differential Mutagenic Potentials of AFB_1_ and ST in MEFs

The mutational frequency analysis of AFB_1_ and ST in MEFs with S9 metabolic activation (Figure 3C) revealed striking differences in their mutagenic capacity, reflecting their distinct metabolic and mutagenicity profiles (vide infra). AFB_1_ exhibited a strong, dose-dependent increase in mutation frequency, with the highest concentration (0.5 µM) showing a pronounced elevation as compared to lower doses (0.1 and 0.3 µM). This result highlights the potent mutagenic capacity of AFB_1_’s reactive metabolites generated through bioactivation.

In contrast, ST displayed only a modest increase in mutation frequency, with no clear dose-dependent trend observed across the tested concentrations (0, 0.05, 0.1, and 0.2 µM). The selected doses for this study were based on prior cytotoxicity assessments, ensuring sufficient cell viability to capture the mutagenic effects of the mycotoxins. AFB_1_ consistently demonstrated a significantly higher mutagenic activity than ST. While both AFB_1_ and ST depend on metabolic activation for their genotoxic effects, AFB_1_ appears to be metabolized more readily into its reactive form, contributing to its stronger mutagenic potential.

### 2.3. Unique Mutational Spectra of Metabolically Activated AFB_1_ and ST

**Figure 4:**
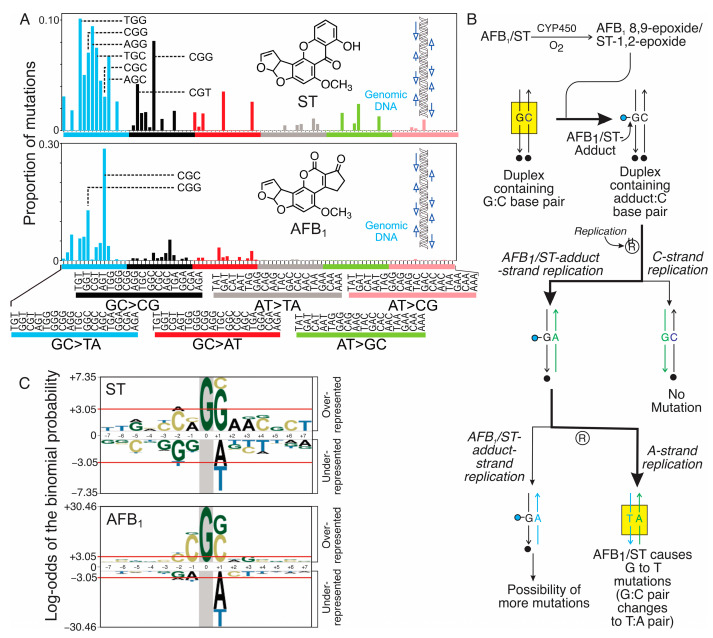
Mutational spectra, a proposed mutational pathway and sequence context preferences of AFB_1_ and ST for mutagenesis. Mutational spectra and probability LOGO (pLOGO) analyses of AFB_1_ (0.5 µM) and ST (0.2 µM) reveal their distinct mutagenic properties. (A) Proportion of mutations caused by AFB_1_ (bottom) and ST (top) across the interrogated areas of the genome. Shown here are background-subtracted mutational spectra. Background spectra are included in Supplementary Material, Figure S2. AFB_1_ predominantly induced GC→TA transversions in specific GC-rich contexts, while ST showed a lower mutation frequency and a broader range of GC→TA 3-base mutation contexts and mutation types. (**B**) Mutation logic diagram of AFB_1_ and ST. CYP450 enzymes convert AFB_1_ and ST into their epoxide forms, which form covalent adducts (shown as blue lollipops) with guanine bases in GC base pairs (a GC pair undergoing adduction is shown in the yellow box). During replication, these adducts can mispair; predominant mispairing is guanine with adenine, resulting in GC→TA transversions. Bold arrows show the main pathway to GC→TA mutations. Parental duplexes are black and marked with black circles under the duplexes. Upon replication (encircled “R” character), strands separate into black/green hybrids, where green denotes the nascent strand. A subsequent round of replication yields a green/blue hybrid, which contains an AT pair (in lower yellow box); this mutation occurs at the site of the original GC pair in the parental (black strands) genome. Alternative pairings may lead to other mutation types. (C) pLOGO analyses of the 15mer sequence contexts surrounding G→T mutations reveal sequence-specific preferences. Both compounds target guanine at the central mutation site (the large G at position 0), with overrepresented flanking nucleotide sequences suggesting unique interaction patterns with DNA. AFB_1_ displays a sharper sequence context bias compared to ST, underscoring its higher mutagenic potency and specificity. Statistical significance thresholds (*p* < 0.05) are indicated by red horizontal lines.

Both AFB_1_ and ST exhibited mutational profiles dominated by GC→TA transversions (Table 1 and blue bars in Figure 4A). While some features of the mutational patterns look similar, for the most part, the patterns are distinct, with a cosine similarity of only ~0.4 between the background-subtracted spectra (see Figure S2 for the background spectra and Figure S3 for the clustering analysis). Given the nearly exclusive propensity of both toxins to damage guanines, we assume that the G→T mutations originate from the polymerase-mediated insertion of adenines opposite the adducted guanines. This mechanism of mutagenesis, termed the “A-rule”, is seen when individual adducts of aflatoxin, as well as many bulky or helix-distorting mutagens, are replicated in vitro and inside living cells [30]. Figure 4B shows a model explaining the pathway underlying the G→T mutations observed from both AFB_1_ and ST. In this model, the guanine of an undamaged GC base pair (in the upper yellow box) becomes an AFB_1_ or ST adduct (the blue lollipops). Replication of the strands containing each lesion results in an adenine being put in opposite the respective adducts; further rounds of replication reveal the emergence of GC→TA mutations (lower yellow box).

Both compounds demonstrated strong sequence-context biases in their mutational patterns, with GC-rich trinucleotide motifs prominently targeted (Figure 4A,C). Interestingly, AFB_1_ displayed a heightened preference for mutation at 5′-CGC-3′ sequences (the underscored G indicates the position in the 3-base context where mutations occur), likely indicative of a unique binding interaction with DNA that precedes alkylation. This characteristic hotspot for G→T mutations was previously observed in the livers of mice treated with AFB_1_ [34]. While ST also predominantly induced G→T mutations, its G→T mutations are much more uniformly distributed among all 16 3-base sequence contexts (Figure 4A, top panel). Moreover, ST induced a more diverse spectrum of base substitutions, with additional contributions evident in GC→CG (black bars), AT→GC (green bars), and GC→AT (red bars) domains, suggesting concomitant oxidative stress or alternative DNA repair pathways.

Probability LOGO (pLOGO) analysis further refined the sequence context distributions around the guanines targeted by both mycotoxins, which become the sites for the G→T mutations (Figure 4C). This analysis provides statistical data on the sequence-specific preferences for mutation by looking at an expanded window of sequences (+/− 7 bases) around the site of each mutation. The results recapitulate well the patterns observed in the trinucleotide mutational spectra (Figure 4A), highlighting the different sequence biases for the two mycotoxins. For AFB_1_, the G→T mutations occur most often in CGC and CGG sequence contexts, whereas for ST, the G→T mutations are mildly enriched in NGG sequence contexts (where N = any base). No longer range (beyond the immediate flanking bases) sequence context preferences were observed for either compound. Moreover, no significantly enriched sequence contexts were found in the pLOGO analysis on control spectra (Figure S4).

**Figure 5:**
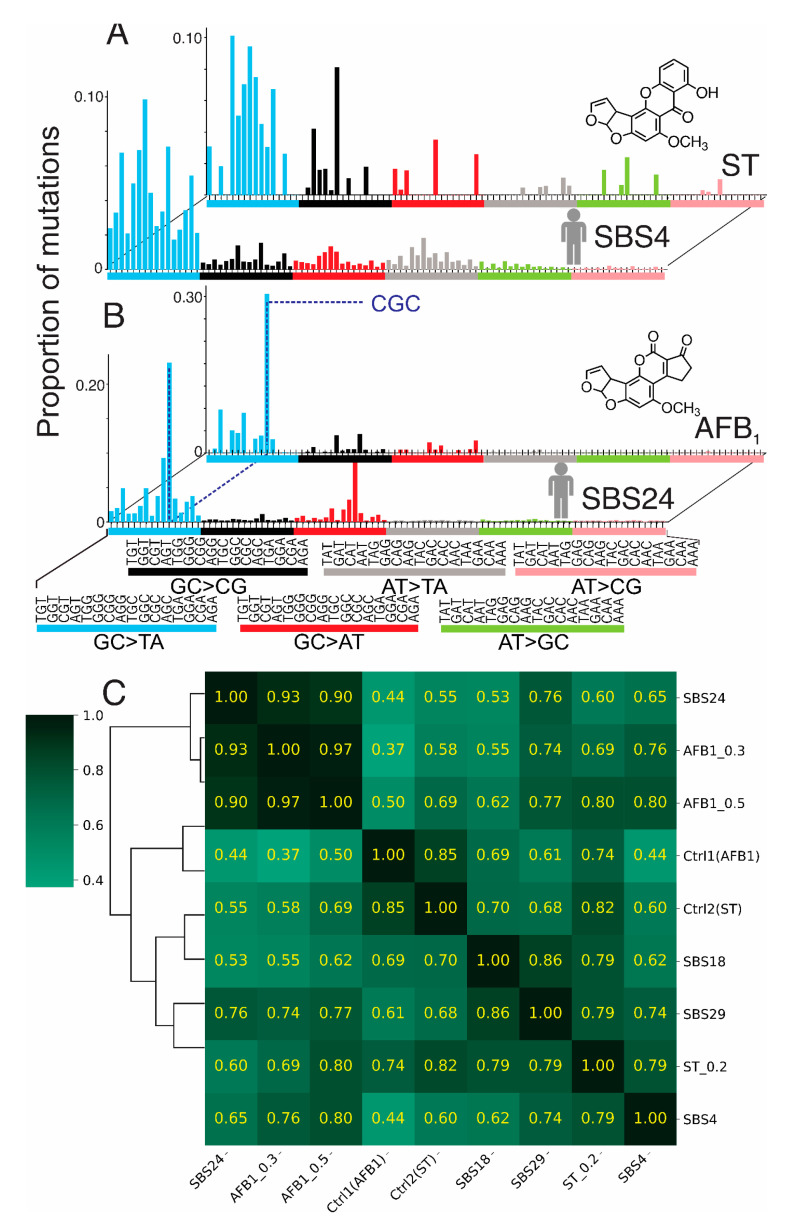
Comparison of mutational spectra of AFB_1_ and ST with computationally derived mutational signatures of human cancers. (**A**) Comparison of mutational spectrum of ST from present work with human mutational signature SBS4, which is common in tobacco-associated cancers. Cosine similarity of these two spectra is 0.79. (**B**) Similar comparison with mutational spectrum of AFB_1_ with human SBS24, which is seen in hepatocellular carcinomas of people from areas of the world where exposure to aflatoxin was likely. (**C**) Cosine similarity matrix comparing the data from present work on ST and AFB_1_ with control spectra (background from vehicle-treated MEFs), human SBS18, human SBS29, human SBS24, and human SBS4. As one example, AFB_1_ at 0.5 µM shows a cosine similarity of 0.9 with hepatocellular carcinoma signature SBS24. The intensity of the green color in each square reflects the level of similarity among elements in the matrix.

Unsupervised clustering using the cosine similarity metric (Figure 5 and Figure S3) aligned the mutational signature of AFB_1_ with COSMIC signature SBS24, which is characteristic of aflatoxin-induced mutagenesis; this signature is enriched for G→T substitutions in certain guanine-rich trinucleotide contexts. AFB_1_ showed a strong concordance with SBS24 (cosine similarity = 0.90–0.93), underscoring its potent guanine-directed mutagenic activity. ST correlated only weakly with SBS24 (cosine similarity = 0.60) and, indeed, shared a higher similarity to the COSMIC signatures SBS18, SBS4, and SBS29 (cosine similarity = 0.79). SBS18 is associated with oxidative damage, whereas SBS4 and SBS29 are both linked to tobacco exposure (smoking and chewing, respectively). Interestingly, ST has been identified as a fungal contaminant in dried tobacco leaves [54,55], and therefore, it is conceivable that the tobacco-associated mutational signatures may reflect an ST-induced mutational process.

## 3. Discussion

AFB_1_ and ST are structurally related toxins, both derived from fungal species. They share a common furofuran motif that is principally responsible for the biological activity of their electrophilic metabolites. Both are known to be potent mutagens and carcinogens, capable of forming bulky DNA adducts that disrupt normal DNA replication and repair processes, ultimately promoting genomic instability and cancer [28,30,42,56,57,58]. While both compounds are activated by epoxidation of their terminal furan rings, this study has revealed several differences in their toxicological properties that suggest that, in the case of ST, features not related to epoxidation contribute significantly to its toxic and genetic effects [4,28,30,59]. This work explored these differences in depth and projects implications for cancer etiology, mutational profiling, and potential public health interventions.

AFB_1_ and ST both require bioactivation via Phase I metabolic enzymes, predominantly cytochrome P450s, to exert their mutagenic effects [18,42]. This activation results in the formation of highly electrophilic intermediates, the AFB_1_-8,9-epoxide and ST-1,2-epoxide, that covalently bond to nucleophilic sites in DNA, particularly guanine residues [43]. It is noteworthy that the biological effects of each epoxide can be mitigated in vivo by Phase II enzymes, including glutathione transferases and epoxide hydrolases [60]. If the aflatoxin epoxide evades destruction, it will react with guanine residues to form its initial adduct, AFB_1_-N7-Gua. This adduct can spontaneously depurinate [61] and its presence in the urine of exposed individuals has become a powerful biomarker of aflatoxin exposure [62]. An alternative fate of DNA-bound AFB_1_-N7-Gua involves hydrolysis of its imidazole ring to form the AFB_1_-FAPY adducts (several isomers exist). Formation of the FAPY adducts, particularly the *Ra* atropisomer where the deoxyglycosidic bond is in the β configuration [27] (often referred to as FAPY-I or FAPY-minor) is the principal pathway to AFB_1_-induced mutations, resulting in the well-documented emergence of guanine-to-thymine (G→T) transversions [28,30,33]. The AFB_1_-FAPY adducts, which are relatively refractory to repair and hence persist for long times in the genome, are likely to be the principal source of aflatoxin’s replication errors [30].

ST shares many of aflatoxin’s properties but exhibits lower reactivity toward guanine residues, likely due to differences in the structure of its reactive intermediates, which lead to the formation of fewer and less stable DNA adducts compared to AFB_1_ [22]. As a result, ST-induced DNA adducts are less abundant, and their G→T mutagenic effects are comparatively moderate. However, ST compensates for this lower efficiency by inducing broader types of mutagenic DNA damage, including oxidative lesions mediated by reactive oxygen species (ROS) [46,47]. This dual mechanism of direct DNA damage and oxidative stress probably contributes to the more diverse mutational spectrum of ST, which includes not only G→T transversions but also thymine-to-cytosine (T→C) and cytosine-to-thymine (C→T) transitions (Table 1). The interplay among these mechanisms underscores the complexity of ST’s biological activity and highlights its potential to cause diverse mutagenic outcomes.

The cytotoxicity profiles of AFB_1_ and ST provide further insights into their distinct biological behaviors. ST is intrinsically more cytotoxic than AFB_1_, likely due to its ability to affect cellular processes beyond those triggered solely by covalent DNA damage [46]. For example, ST’s strong association with ROS generation, as indicated above, provides a clue that oxidative stress may play a significant role in its cytotoxicity. ROS can damage cellular membranes, proteins, and other macromolecules in addition to DNA, leading to widespread cellular dysfunction [47,63,64]. This broader cytotoxic activity may explain why ST causes greater inhibition of cell proliferation at equivalent concentrations compared to AFB_1_, even in the absence of S9.

With regard to mutagenicity, AFB_1_ exhibited a higher dose-dependent mutagenicity than ST, likely driven by its high efficiency in forming guanine adducts that evade repair mechanisms. The persistence of AFB_1_-FAPY adducts through multiple replication cycles makes them highly mutagenic [28,30,36]. Analysis of AFB_1_-induced high-resolution mutational spectra reveals a striking enrichment of G→T transversions, particularly in the guanine-rich trinucleotide contexts CGG and CGC (where the underscored base is the site of mutation). This specificity is likely owed to the property of the AFB_1_ epoxide to interact preferentially with guanines in the underscored 3-base contexts. The specific mutational patterns observed for aflatoxin have repeatedly been observed in different mammalian systems, including mouse liver in vivo [34], human HepG2 and HepaRG liver cell lines [35], and human-induced pluripotent stem cells (hiPSCs) [65]. There is remarkable concordance among the results of these genetic studies, which paint a strikingly consistent picture of the mechanism of aflatoxin-induced mutations and cancer.

ST-induced mutations, on the other hand, lack such pronounced context-specificity. While G→T transversions are prominent in ST’s mutational profile, they are accompanied by a broader spectrum of other substitutions, including T→C and C→T. One factor leading to a broader mutational pattern might be the propensity of ST to induce oxidative DNA lesions in cells [64], in addition to covalent ST-base adducts, thus leading to a multifaceted mechanism of action [66]. Oxidative stress by ST could lead to oxidative lesions such as 7,8-dihydro-8-oxodeoxyguanosine (8-oxo-dG), which mispairs with adenine during replication [46,47,67]. Moreover, oxidative damage to thymine [68] as well as adenine deamination can lead to T→C substitutions [69], mutations that are also seen in ST’s mutational spectrum (Figure 4A). In addition to adverse genetic effects, oxidative stress induced by ST could activate cellular signaling pathways involved in inflammation, apoptosis, and stress responses, further amplifying ST’s cytotoxic and mutagenic effects. These secondary effects may also play a role in the broader range of cancers associated with ST exposure, including colorectal and gastric malignancies [46,47].

An interesting feature of the data may shed light on the mechanism underlying the differences between the respective mutagenicities and toxicities of ST and AFB_1_. As indicated above, ST (without metabolic activation) is more toxic than AFB_1_ in our model system. A straightforward explanation could be its more efficient penetration into the cells. Another intriguing hypothesis, however, is that ST may interact with DNA more favorably than aflatoxin as an intercalating agent. ST (as well as its epoxide) has a completely planar xanthone ring system (Figure 1) that is responsible for its potent ability to intercalate into DNA. If the intercalation event, which precedes the covalent reaction with guanine in the case of the ST-epoxide, is not very sequence-specific, one would expect that ST would form adducts with guanine residues in many different sequence contexts. Supporting this notion, Gopalakrishnan et al. provided evidence to show that the xanthone ring system is intercalated in the solution structure of ST-N7-Gua in DNA [70]; moreover, the bases 5′ and 3′ to the adducted guanine do not appear to affect eventual binding. Based on that result, one would expect that ST would bond with guanines in a relatively context-independent manner, resulting in a relatively uniform distribution of G→T mutations as seen in Figure 4A. Aflatoxin, by contrast, has a cyclopentenone ring, lacking in ST, that is puckered by two adjacent *sp^3^* centers. Because AFB_1_, or more appropriately the AFB_1_-8,9-epoxide, is less planar than ST, one could speculate that it is more discriminating in the sequence contexts into which it will intercalate. The hotspots in which we see AFB_1_ mutations are 5′-CGC-3′ and 5′-CGG-3; these contexts are suggested to be hotspots for binding, which would result in the observed mutational pattern favoring mutations at the underscored guanines in vivo.

The integration of high-resolution mutational spectra with COSMIC mutational signatures provides a valuable framework for understanding the carcinogenic potential of AFB_1_ and ST. AFB_1_’s mutational profile characterized by G→T transversions in guanine-rich contexts that are strongly aligned with COSMIC signature SBS24 makes it likely that human SBS24 is due to environmental exposure to aflatoxin [30,34,65]. This alignment underscores AFB_1_’s specificity as a guanine-targeted mutagen and its well-established role in the etiology of hepatocellular carcinoma [31,71,72].

The ST spectrum shares some similarities with SBS24 but also exhibits stronger features that align with COSMIC signature SBS18, which is associated with oxidative stress-related mutagenesis [73]. This overlap highlights ST’s dual mechanisms of mutagenicity, combining direct adduct formation with ROS-mediated damage. The partial alignment of both AFB_1_ and ST with COSMIC signature SBS4 (Figure 5C), linked to bulky DNA adducts formed by environmental carcinogens such as tobacco, further emphasizes their shared pathways of adduct-induced mutagenesis [65,73]. These findings provide important insights into both the distinct and overlapping mechanisms of AFB_1_ and ST and their relevance to human cancers.

Lastly, this study was motivated by two issues. First, AFB_1_ and ST are important environmental toxins, and it was timely to use recently developed tools to delve deeply into their genetic effects. Second, however, we saw the opportunity to compare the genetic effects of two natural products that differ in from one another in subtle ways. In the interest of further mechanistically informative structure–activity studies, the aflatoxin family of molecules offers many opportunities for detailed study. As a few examples, aflatoxins M_1_, G_1_, Q_1_, and P_1_ have structural features that likely would affect the interaction of their respective epoxides with DNA, and they would possibly display distinctive mutational spectra.

## 4. Conclusions

The research described here defined the distinct mutagenic landscapes of structurally similar mycotoxins, AFB_1_ and ST. Through high-resolution mutation analysis, unique genomic fingerprints were established, and these fingerprints could be future biomarkers aiding the fields of risk assessment and cancer prevention. The increasing pressures from climate change and global food contamination further highlight the need for improved mechanistically-informed public health policies. While the carcinogenic properties of AFB_1_ are well recognized and closely regulated, ST and other members of the aflatoxin family are markedly less governed, underscoring a possible lack of sufficient awareness regarding their dangers. Modern genomic technologies will help further refine toxicological model systems and support the precision prevention strategies. Lastly, AFB_1_ and ST serve as critical models for understanding the interplay among DNA adduct formation, oxidative damage, and mutagenesis.

*Dedication*: We are pleased to have this paper included in the series honoring the many contributions of our colleague, Professor John D. Groopman, to mycotoxin research. His work, especially in the biomarker, cancer epidemiology, and chemo-prevention fields, serves as a model and inspiration for future generations of toxicologists.

## 5. Materials and Methods

### 5.1. Cell Culture and Growth Inhibition Assay

*Gpt*Δ C57BL/6J MEFs, confirmed mycoplasma-free by PCR, were established and maintained as previously described [52]. The effects of AFB_1_ (Sigma-Aldrich, St Louis, MO, USA) and ST (kindly provided by George H. Buchi (MIT)), with purity confirmed by NMR as shown in the Supplementary materials Figure S1 on cell growth inhibition, were assessed. For both compounds, 2.5 × 10^4^ cells per well were seeded in 6-well plates containing growth media (high glucose DMEM containing GlutaMAX (Life Technologies, Carlsbad, CA, USA) supplemented with 10% FBS (VWR Scientific Products, Pittsburgh, PA, USA), 100 IU/mL penicillin, 100 µg/mL streptomycin (Sigma-Aldrich, St Louis, MO, USA), and 1 mM sodium pyruvate (Life Technologies, Carlsbad, CA, USA)) and incubated overnight in a humidified atmosphere containing 5% CO_2_. The cells were then treated with either 0–1 µM AFB_1_, 1% *v*/*v* Aroclor 1254-induced S9 fraction (Fisher Scientific, Pittsburgh, PA, USA), and 1 mM NADPH (Sigma-Aldrich, St Louis, MO, USA), or 0–0.5 µM ST with 2% *v*/*v* Aroclor 1254-induced S9 and 2 mM NADPH in 1X HBSS (Life Technologies, Carlsbad, CA, USA), and incubated for 3 h, after which the cells were washed with 1X PBS (pH 7.4, Life Technologies, Carlsbad, CA, USA) before fresh growth media was added. Following a 48 h recovery period in the incubator, cell growth was assessed by trypsinizing the MEFs and counting them using the Vi-CELL XR cell viability analyzer (Beckman Coulter, Brea, CA, USA).

### 5.2. Gpt Mutation Assay

MEFs were seeded in 100-mm tissue culture plates at a density of 5 × 10^5^ cells per plate and allowed to adhere overnight. Following this incubation, the growth medium was replaced with treatment 1X HBSS solutions containing either 0–0.2 µM AFB_1_ combined with 1% *v*/*v* Aroclor 1254-induced S9 fraction and 1 mM NADPH, or 0–0.5 µM ST with 2% S9 and 2 mM NADPH. The MEFs were exposed to these treatments for 3 h, after which they were washed with 1X PBS to remove the treatment solution and subsequently allowed to recover in fresh growth media for 48 h, following the established growth inhibition assay protocol. After the recovery period, the cells were harvested and washed twice with 1X PBS. Genomic DNA was isolated from 2 × 10^6^ cells per treatment group using the RecoverEase DNA Isolation Kit (Agilent Technologies, Santa Clara, CA, USA). The λ-EG10 phage was then packaged in vitro from the extracted genomic DNA using the Transpack Packaging Extract (Agilent Technologies, Santa Clara, CA, USA) and transfected into *Escherichia coli* YG6020, which expresses Cre-recombinase, thereby generating a 6.4 kb plasmid encoding both the *gpt* and chloramphenicol acetyltransferase genes. The transfected *E. coli* were cultured on selective media containing 25 µg/mL chloramphenicol (CHL, Sigma-Aldrich, St Louis, MO, USA) and 25 µg/mL 6-thioguanine (6-TG, Sigma-Aldrich, St Louis, MO, USA), or on media containing only CHL. Confirmation of 6-TG resistance was achieved through the regrowth of colonies on plates supplemented with both CHL and 6-TG. The mutational frequency for each treatment group was subsequently calculated as the ratio of total 6-TG-resistant colonies to the average number of CHL-resistant colonies, consistent with methodologies outlined in prior studies [50,52,53].

### 5.3. Duplex Sequencing (High-Resolution Mutational Spectrometry)

Genomic DNA for duplex sequencing (DS) libraries was extracted from MEFs that had been treated with AFB_1_ or ST using the same treatment protocol as in the *gpt* assay. The DNA extraction was carried out using the dNeasy Blood and Tissue Kit (Qiagen, Valencia, CA, USA), following the manufacturer’s instructions. Approximately 1 µg of purified genomic DNA was then utilized to construct the DS libraries with the Mouse Mutagenesis Kit (TwinStrand Biosciences, Seattle, WA, USA). This kit employs specially designed probes that hybridize to 20 distinct genomic regions, each measuring 2.4 kb, strategically chosen to encompass intronic and intergenic areas, thereby minimizing selection bias. Sequencing was conducted on an Illumina NovaSeq 6000 DNA sequencer with an S4 flow cell, utilizing a 150 bp paired-end protocol, supported by the MIT BioMicro Center. Detailed genomic coordinates for the targeted regions can be found in the Supplementary Materials, Table S2.

### 5.4. Data Processing

The data generated from the TwinStrand DuplexSeq assay were processed using the TwinStrand DuplexSeq Mutagenesis App (v4.5.0) via the DNANexus platform. Mutation-position files (.mut) produced by this pipeline were further analyzed using custom Python scripts available on GitHub (see Data Availability Statement). For each sample, a unique list of mutations was compiled, ensuring that a mutation at any specific genomic location was counted only once. Using this list, trinucleotide mutational spectra were constructed and normalized by dividing the observed mutation frequency for each trinucleotide context by the frequency of that context within the target genomic sequence (Figure S5). To compare the mutational spectra, cosine similarity was calculated against spectra generated within this study and the single base substitution (SBS) mutational signatures from the COSMIC v3.4 repository. Additionally, pLOGO plots were generated by extracting 15mer sequence contexts (7 bases upstream and downstream of each G→T mutation) and analyzing them with the Schwartz pLOGO tool (https://plogo.uconn.edu/; version accessed on 7 February 2025).

### 5.5. Statistical Analysis

All data are presented as mean ± standard deviation. Statistical analyses were performed using GraphPad Prism 8. Differences between groups were evaluated using the two-tailed Student’s *t*-test. Statistical significance was defined as a *p*-value < 0.05. Each experiment was conducted in triplicate to ensure reproducibility, and the results were subjected to rigorous statistical validation to confirm reliability and accuracy.

## Figures and Tables

**Table 1:** Relative frequencies of point mutation types in mutational spectra corresponding to AFB_1_-treated and ST-treated MEFs with metabolic activation (spectra shown in Figure 4), and corresponding vehicle controls (spectra shown in Supplementary Materials, Figure S2). Both AFB_1_ and ST data reflect background-subtracted spectra. An expanded version of this Table is in Supplementary Materials (Table S1).

Substitution Type	AFB_1_ (%)	AFB_1_-Control (%)	ST (%)	ST- Control (%)
**Transitions**				
**GC**→**AT**	10.3	27.9	9.6	26.7
**AT**→**GC**	0.0	11.6	5.9	11.6
**Transversions**				
**GC**→**TA**	74.0	32.4	60.8	30.4
**GC**→**CG**	14.3	9.8	19.1	10.3
**AT**→**CG**	0.5	9.6	1.4	10.2
**AT**→**TA**	0.8	8.7	3.1	10.8
**Percentage of** **all substitutions**	100	100	100	100

## Data Availability

The sequencing data files referenced in the present work have been uploaded to the Sequence Read Archive (SRA) repository of the National Center for Biotechnology Information (NCBI) as the BioProject PRJNA1217835 (http://www.ncbi.nlm.nih.gov/bioproject/1217835). The data analysis and visualization Python scripts are available on GitHub (https://github.com/essigmannlab/AFB1_ST_spectra/).

## Supplementary Materials

The following supporting information can be downloaded at: https://www.mdpi.com/article/10.3390/toxins17030112/s1, Figure S1: ^1^H-NMR and ^13^C-NMR spectra of sterigmatocystin in DMSO-d_6_ [74,75]; Figure S2: Mutational spectra of vehicle controls; Figure S3: Unsupervised clustering and cosine similarity matrix of background-subtracted spectra of AFB_1_ and ST; Figure S4: pLOGO probability distribution of G→T mutations in control spectra; Figure S5: Trinucleotide frequencies for sequences covered by TwinStrand mouse probes; Table S1: Relative frequencies of point mutation types in mutational spectra; Table S2: Genomic coordinates of hybrid-captured regions analyzed by TwinStrand protocol.
