# Supplementary material for: Structurally Similar Mycotoxins Aflatoxin B1 and Sterigmatocystin Trigger Different and Distinctive High-Resolution Mutational Spectra in Mammalian Cells
Source: Toxins (Basel). 2025 Feb 27;17(3):112. doi: 10.3390/toxins17030112 (PMC11945433; doi:10.3390/toxins17030112)
Supplement: Supplementary file 1 [file toxins-17-00112-s001.zip › toxins-3492410-supplementary.pdf]

# Supporting Information

# Structurally Similar Mycotoxins Aflatoxin $B_1$ and Sterigmatocystin Trigger Different and Distinctive High-Resolution Mutational Spectra in Mammalian Cells

**Contents:**


1. **Figure S1.** $^1$H-NMR and $^{13}$C -NMR spectra of sterigmatocystin in DMSO-$d_6$ [74,75].
2. **Figure S2.** Mutational spectra of vehicle controls.
3. **Figure S3.** Unsupervised clustering and cosine similarity matrix of background-subtracted spectra of $AFB_1$ and ST.
4. **Figure S4.** pLOGO probability distribution of G→T mutations in control spectra.
5. **Figure S5.** Trinucleotide frequencies for the sequences covered by the TwinStrand mouse probes.
6. **Table S1**. Relative frequencies of point mutation types in mutational spectra.
7. **Table S2.** The genomic coordinates of hybrid-captured regions analyzed by the TwinStrand protocol.

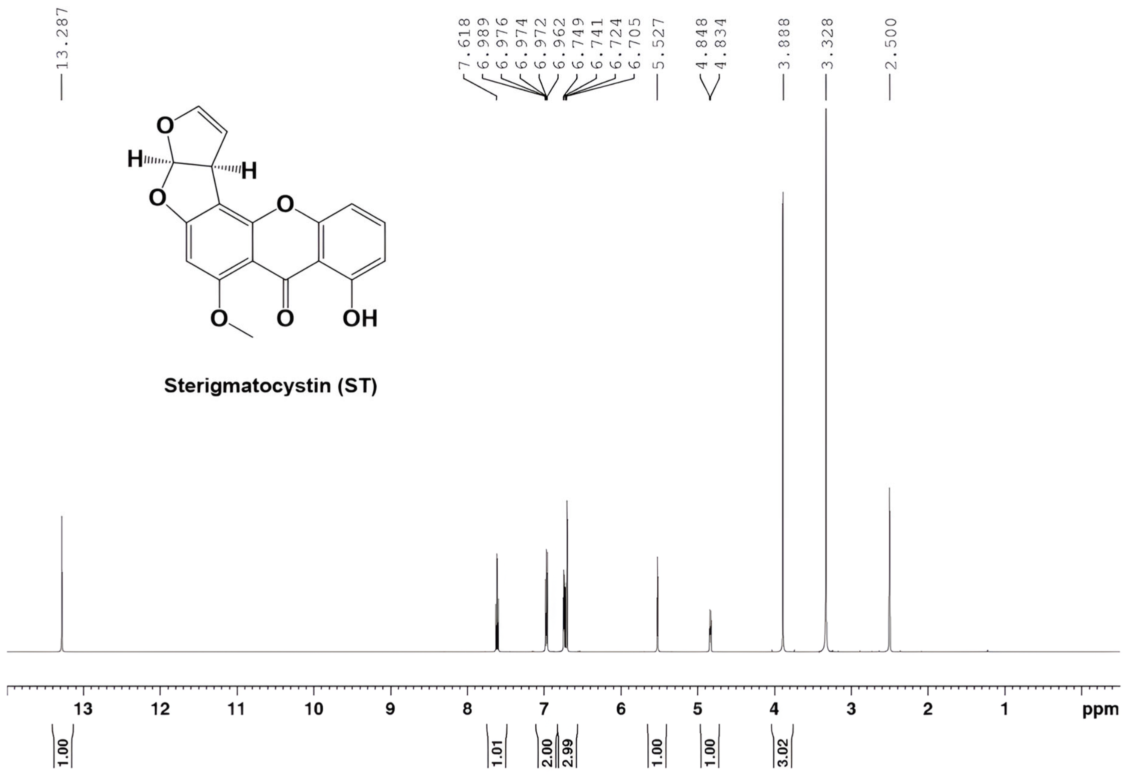

13.287
7.618
6.989
6.976
6.974
6.972
6.962
6.749
6.741
6.724
6.705
5.527
4.848
4.834
3.888
3.328
2.500
Sterigmatocystin (ST)
13 12 11 10 9 8 7 6 5 4 3 2 1 ppm
1.00
1.01
2.00
2.99
1.00
1.00
3.02


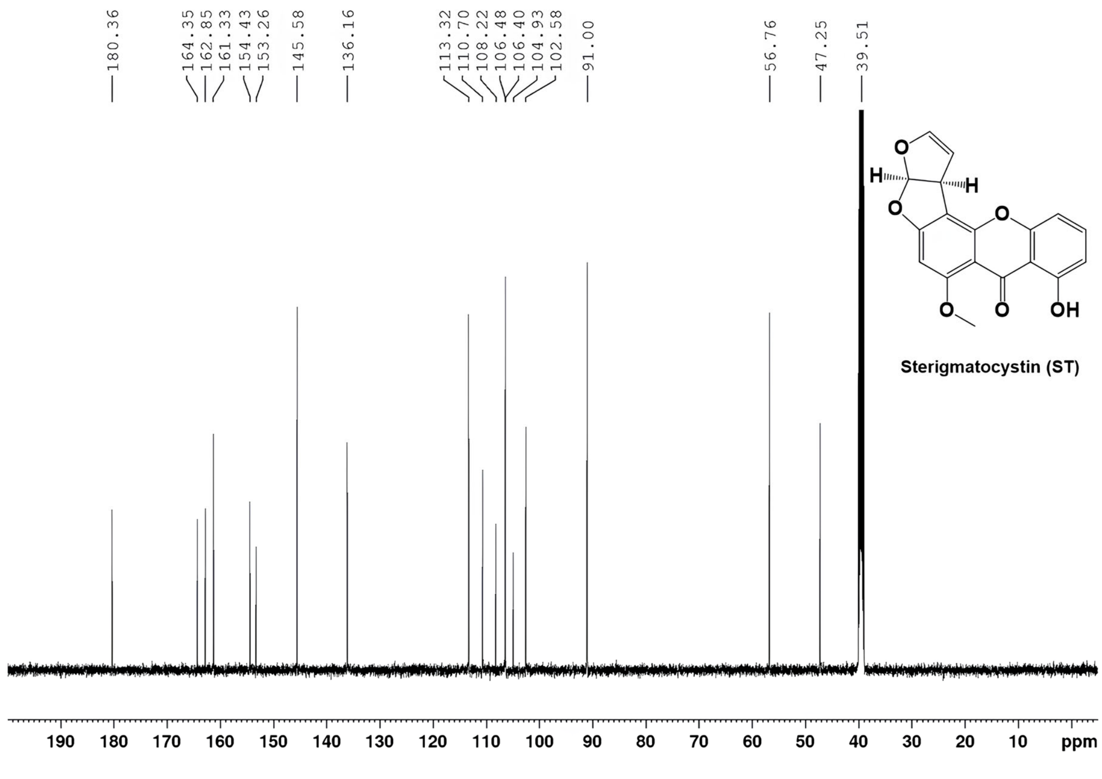

180.36
164.35
162.85
161.33
154.43
153.26
145.58
136.16
113.32
110.70
108.22
106.48
106.40
104.93
102.58
91.00
56.76
47.25
39.51
Sterigmatocystin (ST)
190 180 170 160 150 140 130 120 110 100 90 80 70 60 50 40 30 20 10 ppm

**Figure S1.** $^1$H-NMR and $^{13}$C-NMR spectra of sterigmatocystin (ST) in DMSO-$d_6$. ST in this study was obtained from Professor George H. Buchi of the Massachusetts Institute of Technology and characterized by NMR spectroscopy (on a 500 MHz spectrometer acquired at 500 MHz for $^1$H and 126 MHz for $^{13}$C). Chemical shifts are reported in ppm relative to residual solvent peaks (DMSO-$d_6$: $^1$H, 2.50; $^{13}$C, 39.5). Peak multiplicity patterns are reported as singlet (s), doublet (d), triplet (t), multiplet (m), and doublet of triplet (dt) for $^1$H NMR data. The $^1$H and $^{13}$C NMR peak pattern matches with the previously reported spectra [74,75].

Peaks assignment:

$^1$H NMR (500 MHz, DMSO-$d_6$) δ 13.37-13.22 (s, 1H), 7.68-7.56 (t, 1H), 7.04-6.91 (m, 2H), 6.78-6.74 (m, 1H), 6.74-6.71 (d, 1H), 6.71-6.68 (s, 1H), 5.63-5.42 (t, 1H), 4.97-4.73 (dt, 1H), 4.00-3.76 (s, 3H).

$^{13}$CNMR (126 MHz, DMSO-$d_6$) δ 180.4, 164.4, 162.9, 161.3, 154.4, 153.3, 145.6, 136.2, 113.3, 110.7, 108.2, 106.5, 106.4, 104.9, 102.6, 91.0, 56.8, 47.3.

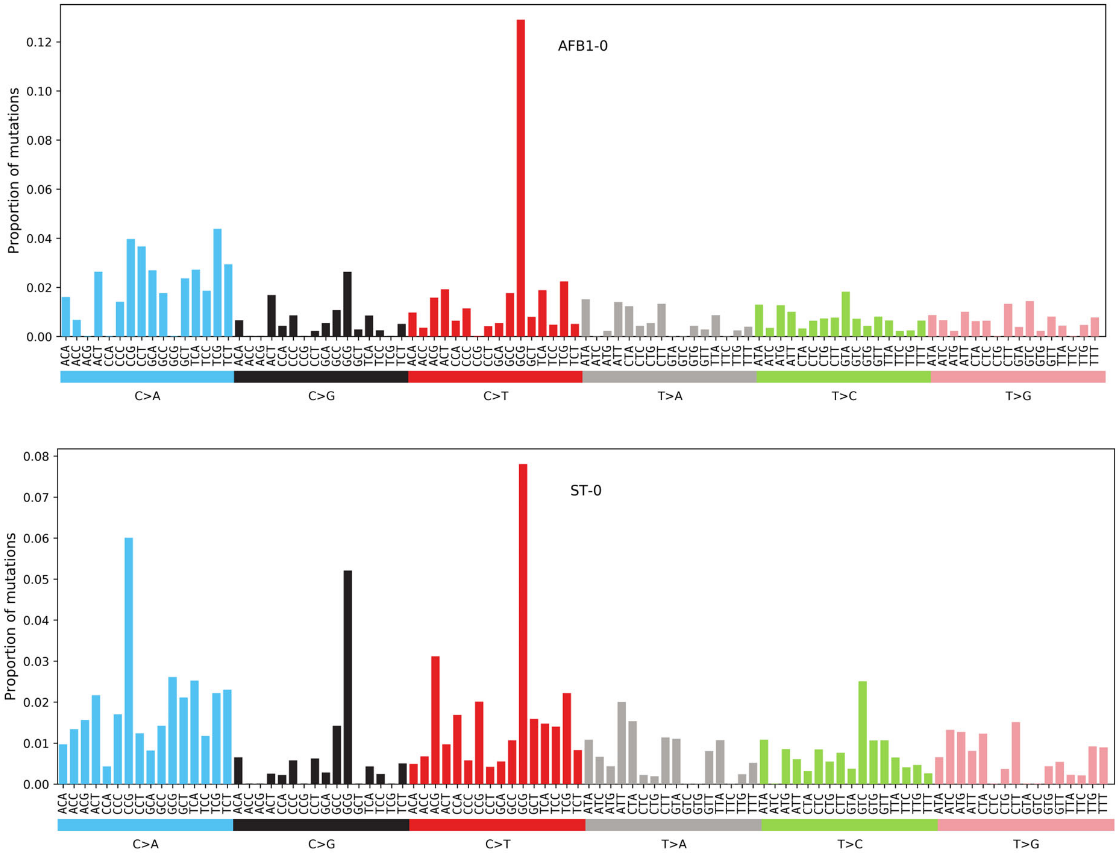


**Figure S2.** Mutational spectra of vehicle controls. High resolution mutational spectra (HRMS) obtained from duplex consensus sequencing of DNA from MEFs exposed to the S9 metabolic activation cocktail but without any toxin. The top spectrum is the control spectrum for the aflatoxin $B_1$ ($AFB_1$) treatments, the bottom spectrum is the control spectrum for the sterigmatocystin (ST) treatments. The HRMS are plotted in three-base contexts, 5′-NXN-3′, where the mutated base (X) is presented along with its neighboring bases (N). Both spectra have been normalized to indicate proportion of mutations per trinucleotide sequence context. This representation indicates the proportion of mutations when assuming an equal abundance of each trinucleotide sequence context in the mouse genome.

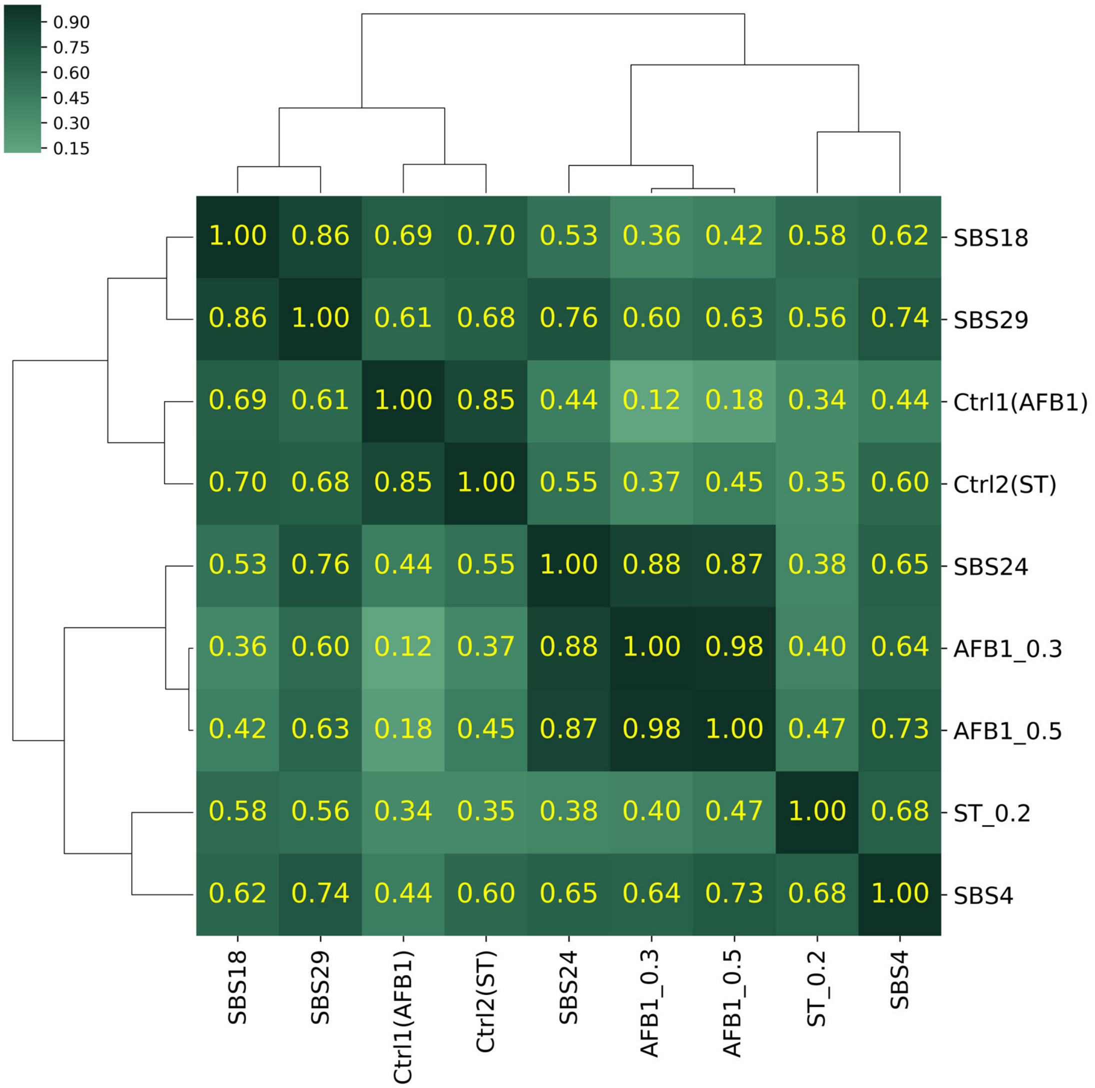


**Figure S3.** Unsupervised clustering and cosine similarity matrix of background-subtracted spectra of $AFB_1$ and ST. A clustering analysis using the cosine similarity metric has been performed using the background-subtracted spectra of $AFB_1$ (at 0.3 µM and 0.5 µM) and ST (at 0.2 µM). The analysis also includes the control spectra, as well as the most similar human mutational signatures (SBS4, SBS18, SBS24, SBS29) from the COSMIC database.

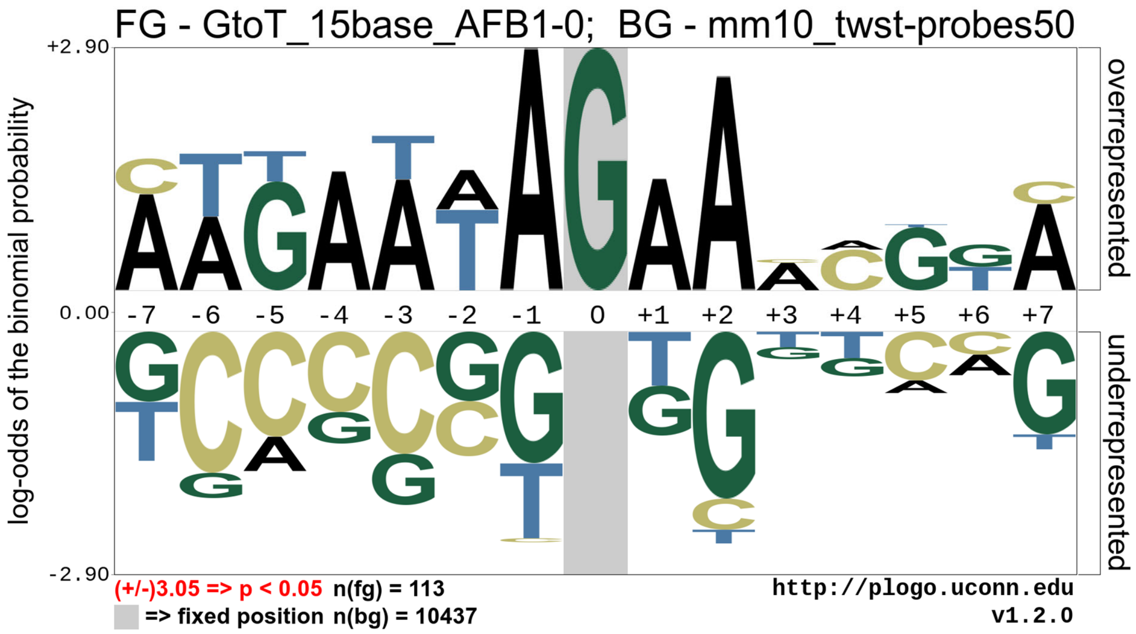


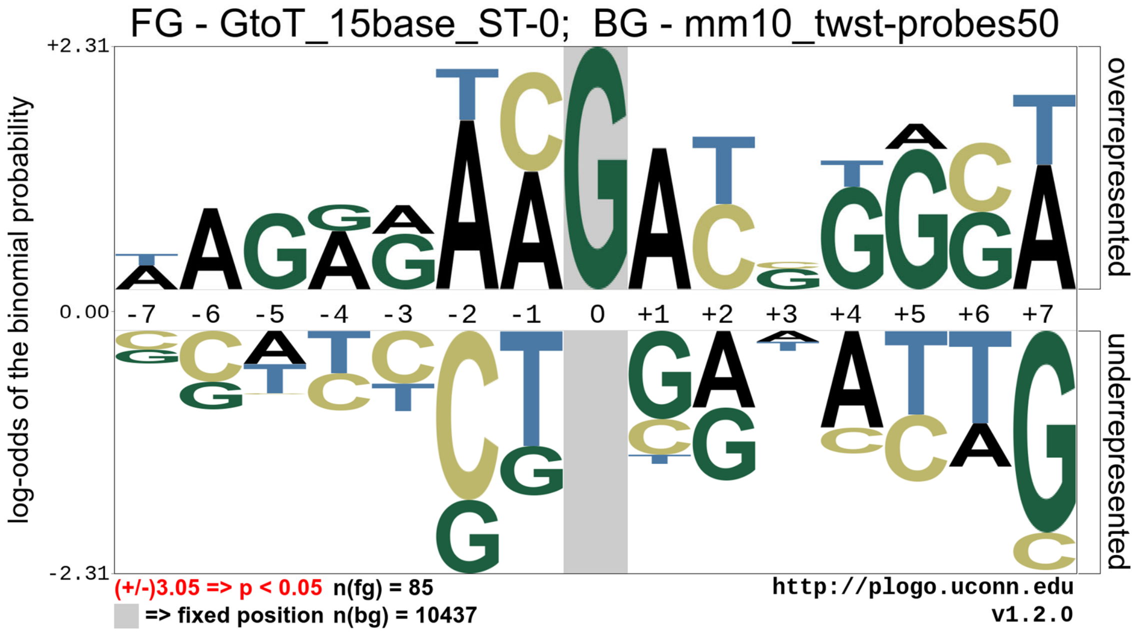


**Figure S4.** pLOGO probability distribution of G→T mutations in control spectra. pLOGO analysis was performed for the distribution of G→T mutations in the control sample corresponding to the $AFB_1$ treatments (top diagram) and ST treatments (bottom diagram). As the log-odds of the binomial probability does not reach 3.05 (which corresponds to a significant $p < 0.05$) for any of the positions investigated around the central G, no sequence-context dependent pattern can be observed for the G→T mutations from either of the two control spectra.

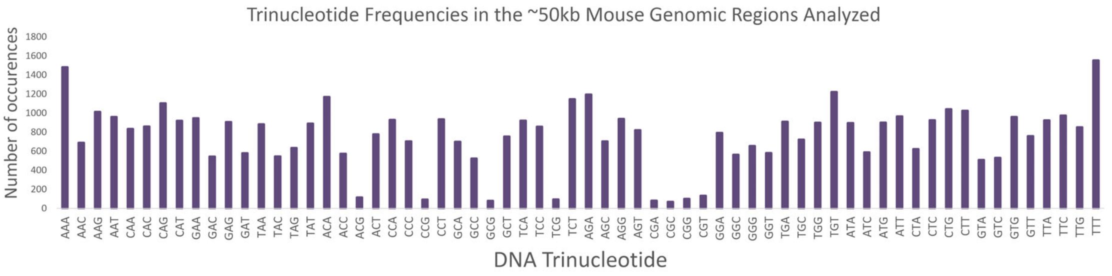


**Figure S5.** Trinucleotide frequencies for the sequences covered by the TwinStrand mouse probes. The distribution of DNA trinucleotide sequences across the ~50kb of mouse genome probed by the TwinStrand procedure is indicated. These frequencies are used to normalize all mutational spectra reported in this study.

**Table S1.** Relative frequencies of point mutation types in the mutational spectra corresponding to vehicle controls, $AFB_1$-treated, and ST-treated MEFs with metabolic activation, before and after subtracting the contribution of their corresponding background (vehicle control) spectrum.

| Substitution type | $AFB_1$-Control (%) | $AFB_1$ before backgr. subtr. (%) | $AFB_1$ after backgr. subtr. (%) | ST-Control (%) | ST before backgr. subtr. (%) | ST after backgr. subtr. (%) |
|---|---|---|---|---|---|---|
| **Transitions** | | | | | | |
| **GC→AT** | 27.9 | 19.4 | 10.3 | 26.7 | 22.2 | 9.6 |
| **AT→GC** | 11.6 | 2.4 | 0.0 | 11.6 | 7.9 | 5.9 |
| **Transversions** | | | | | | |
| **GC→TA** | 32.4 | 59.9 | 74.0 | 30.4 | 46.8 | 60.8 |
| **GC→CG** | 9.8 | 13.3 | 14.3 | 10.3 | 12.8 | 19.1 |
| **AT→TA** | 8.7 | 2.8 | 0.8 | 10.8 | 5.5 | 3.1 |
| **AT→CG** | 9.6 | 2.1 | 0.5 | 10.2 | 4.9 | 1.4 |
| **Percentage of all substitutions** | 100 | 100 | 100 | 100 | 100 | 100 |

**Table S2.** The genomic coordinates (mouse mm10 genome) of the hybrid-captured regions in the TwinStrand protocol using the panel mouse-muta-v1.0.

| Chromosome | Start nucleotide | End nucleotide | Type of region | Gene |
|---|---|---|---|---|
| **1** | 69304217 | 69306617 | intergenic | N/A |
| **1** | 155235938 | 155238338 | genic | Xpr1, intron 2 |
| **2** | 50833175 | 50835575 | intergenic | N/A |
| **3** | 109633160 | 109635560 | intergenic | N/A |
| **4** | 96825280 | 96827680 | intergenic | N/A |
| **5** | 18210612 | 18213012 | genic | Gnat3, intron 5 |
| **6** | 119170706 | 119173106 | genic | Cacna1c, intron 1 |
| **7** | 142683053 | 142685453 | genic | Kcnq1, intron 1 |
| **8** | 43954521 | 43956921 | intergenic | N/A |
| **9** | 28648072 | 28650472 | genic | Opcml, intron 3 |
| **10** | 21442014 | 21444414 | intergenic | N/A |
| **11** | 37934364 | 37936764 | intergenic | N/A |
| **12** | 80601002 | 80603942 | genic | Galnt16, intron 2 |
| **13** | 74030071 | 74032471 | intergenic | N/A |
| **14** | 13076171 | 13078571 | intergenic | N/A |
| **15** | 66779762 | 66782162 | genic | Ccn4, intron 2 |
| **16** | 72381580 | 72383980 | intergenic | N/A |
| **17** | 94009028 | 94011428 | intergenic | N/A |
| **18** | 81262078 | 81264478 | intergenic | N/A |
| **19** | 4618813 | 4621213 | genic | Pcx, intron 2 |
